# White-tailed deer browse on an invasive shrub with extended leaf phenology meets assumptions of an apparent competition hypothesis

**DOI:** 10.1093/aobpla/plx006

**Published:** 2017-02-13

**Authors:** Kylie L. Martinod, David L. Gorchov

**Affiliations:** Department of Biology, Miami University, Oxford, OH 45056, USA

**Keywords:** Crude protein, herbivory, *Lonicera maackii*, *Odocoileus virginianus*, Ohio USA

## Abstract

It has been hypothesized that invasive plant species with extended leaf phenology (ELP) elevate generalist herbivore populations, increasing herbivory on native plants (apparent competition). This hypothesis assumes that consumption of the invasive is associated with periods of ELP, the invasive is a major component of the herbivore’s diet, and that it is more nutritious than native plants during periods of ELP. We tested these assumptions by estimating the proportion of the white-tailed deer diet comprised of *Lonicera maackii*, an invasive shrub with ELP, quantifying the seasonal pattern of deer browse on this invasive shrub, and comparing its nutritional quality to leafless woody stems. In the Miami University Natural Areas in southwest Ohio we quantified the frequency of leafy twigs of woody species 0.3–2.1 m high in three habitats (deciduous forest, *Juniperus virginiana* forest, and forest-field edge). Monthly we quantified deer browse on marked *L. maackii* twigs, and estimated the mass of leaf and stem tissue consumed with allometric relationships using diameter and length of unbrowsed twig portions. We estimated the percent of the annual deer diet comprised of *L. maackii* by dividing the sum of these estimates by the product of deer abundance (estimated by pellet-based distance sampling) and consumption estimates from the literature. Crude protein of *L. maackii* stems and leaves was determined by C:N analyser. In each habitat the frequency of *L. maackii* was greater than all other woody species combined. We estimated *L. maackii* comprised 14–47 % of the annual deer diet. Deer browsed *L. maackii* each month, but consumption was high in early spring and late summer. Crude protein of leafy twigs of *L. maackii* in early spring was 12.9 %, much higher than leafless twigs of native species on-site. These findings support the assumptions of the hypothesis that invasive plants with ELP impact native plants via deer-mediated apparent competition.

## Teaser

We investigated whether white-tailed deer browse on the invasive shrub, Amur honeysuckle, supports key assumptions of the hypothesis that invasives with extended leaf phenology (ELP) impact natives via “apparent competition,” elevating the impact of a shared herbivore. We found that deer browse on honeysuckle was particularly high in early spring, a time of protein scarcity. The leafy twigs of honeysuckle in early spring were higher in protein than leafless twigs of native trees. We estimated that consumption of honeysuckle twigs accounted for 14-47% of the annual food consumption of deer in the study site. These findings show that the hypothesis of apparent competition is compelling.

## Introduction

Impacts of invasive plants on native plants have been demonstrated in many systems, and generally attributed to resource competition or changes in ecosystem processes (Vilà *et al.* 2011; Gioria and Osborne 2014, Jauni and Ramula 2015). Less well studied is the potential impact of invasive plants on native plants via apparent competition, where the negative interaction is indirect, a consequence of both species interacting with a shared enemy, e.g. a predator (Holt 1977, Connell 1990). However, White *et al.* (2006) reviewed several studies illustrating apparent competition or other indirect interactions involving invasive species.

Apparent competition can be food-mediated (trophic), where one species elevates the abundance or feeding activity of the predator, or habitat-mediated (non- trophic), where the species provides a refuge or some other non-food resource that elevates the impact of the predator on the other (White *et al.* 2006, Orrock *et al.* 2010). For plant invasions in temperate forests, several studies report support for non-trophic apparent competition, specifically greater predation on seeds of native species by rodents in areas with cover of non-native shrubs (Meiners 2007, [Bibr plx006-B16][Bibr plx006-B5], but see Mattos *et al.* 2013). The only evidence for food-mediated apparent competition comes from Orrock *et al.*’s (2015) finding that the fruits of the invasive shrub, *Lonicera maackii*, increased the negative effect of rodents on native plant species richness. The potential for large-bodied, generalist grazers and browsers to affect food-mediated apparent competition between invasive and native plants due to mediate apparent competition has been hypothesized (Smith and Hall 2015), but not tested.

Population densities of ungulates, including deer, are elevated compared to historical densities in many parts of the world, with well-documented negative effects on native forest plants (reviewed by Côté *et al.* 2004). Among the major factors implicated in these increases in deer densities is increased availability of forage (Côté *et al.* 2004), frequently attributed to forest management practices or landscape structure (agriculture, increased edge or successional habitat) (reviewed by Côté *et al.* 2004, see also Miyashita *et al.* 2008, Hurley *et al.* 2012). Increased food availability is one reason white-tailed deer (*Odocoileus virginianus*), hereafter ‘deer,’ have surpassed densities preceding European settlement throughout the Eastern and Midwestern USA (McCabe and McCabe 1997).

Food availability for deer might also be elevated by plant invasions in deciduous forests. If an invasive species provides a food resource at a time of year critical to the consumer, this provides a scenario for apparent competition (White *et al.* 2006). For herbivores in temperate areas, a crucial time is the transition from winter to spring; they are affected if forage is scarce or of low quality (Moen 1978). White-tailed deer select foods rich in protein in spring and summer (Berteaux *et al.* 1998, Dostaler *et al.* 2011), particularly leaves, which have more nitrogen than leafless twigs (Mattson 1980; Table 1), although leafless woody twigs are still important foods for white-tailed deer (Tripler *et al.* 2002). When spring comes early (i.e. new stems and leaves expand earlier than usual) pregnant deer, and fawns from these does, have higher survival, as does recover more quickly from winter starvation resulting in larger and stronger fawns (Moen 1978, Pekins *et al.* 1998). Smith (2013) hypothesized that the same positive effect on does and fawns is caused by the extended leaf phenology (ELP) exhibited by many plants invasive in forests of eastern North America (Harrington *et al.* 1989, Fridley 2012). In this hypothesis, invasives with ELP elevate deer carrying capacity and thus deer impacts on native plants via apparent competition (Smith 2013). Motivated by this hypothesis, Smith and Hall (2015) modeled the interaction among an invasive plant, a native plant, and a shared herbivore, and showed that a longer growing season (ELP) for the invasive expanded the range of parameter values where it suppresses the native via apparent competition.

**Table 1.** Percent nitrogen (%N) and/or crude protein (CP) of *L. maackii* from this study and of other woody species from literature. The CP values in parentheses are the %N values from literature that we multiplied by 6.25. Nutritional quality results of *L. maackii* for winter stems, spring twigs, and spring leaves were from a carbon-nitrogen analyser, and spring leafy stems were based on a weighted average. Everitt and Gonzalez (1981) analysed 34 white-tailed deer food plants for %N of leaves and ends of twigs using the Kjeldahl method and then multiplied by 6.25. Reich et al. (1998) estimated leaves of different functional using microKjeldahl digestion techniques. Ordonez and Olff (2013) is a review that reported leaf N content of 2448 native and 961 invasive species from other studies; we include those species that were present in our quadrats. Tripler et al. (2002) determined the mean N content of apical stems of saplings of nine tree species that dominated a conifer-hardwood forest using a CHN combustion analyser.

Species or Functional group	Leaf, stem, or both	Season	CP	%N	Source
*Lonicera maackii*	Stem	Winter	10.0	1.60	This study
*Lonicera maackii*	Stem	Spring	5.4	0.87	This study
*Lonicera maackii*	Leaves	Spring	14.0	2.24	This study
*Lonicera maackii*	Both	Spring	12.9	2.07	This study
Woody	Both	Spring	17.4		Everitt and Gonzales (1981)
Woody	Both	Summer	15.0		Everitt and Gonzales (1981)
Woody	Both	Fall	15.7		Everitt and Gonzales (1981)
Woody	Both	Winter	13.9		Everitt and Gonzales (1981)
Forbs	Both	Spring	13.8		Everitt and Gonzales (1981)
Forbs	Both	Summer	14.6		Everitt and Gonzales (1981)
Forbs	Both	Fall	15.3		Everitt and Gonzales (1981)
Forbs	Both	Winter	17.1		Everitt and Gonzales (1981)
Forbs	Leaves	Growing	(22.1)	3.54	Reich et al. (1998)
Deciduous shrub	Leaves	Growing	(13.0)	2.08	Reich et al. (1998)
Evergreen shrub	Leaves	Growing	(9.9)	1.58	Reich et al. (1998)
Deciduous broad-leaf tree	Leaves	Growing	(13.9)	2.22	Reich et al. (1998)
Evergreen broad-leaf tree	Leaves	Growing	(9.4)	1.50	Reich et al. (1998)
Deciduous needle-leaf tree	Leaves	Growing	(11.9)	1.90	Reich et al. (1998)
Evergreen needle-leaf tree	Leaves	Growing	(7.3)	1.16	Reich et al. (1998)
*Acer negundo*	Leaves	Growing	(15.6)	2.5	Ordonez and Olff (2013)
*Acer nigrum*	Leaves	Growing	(15.6)	2.5	Ordonez and Olff (2013)
*Acer saccharum*	Leaves	Growing	(13.8)	2.1	Ordonez and Olff (2013)
*Celastrus orbiculatus*	Leaves	Growing	(16.9)	2.7	Ordonez and Olff (2013)
*Celtis occidentalis*	Leaves	Growing	(15.0)	2.4	Ordonez and Olff (2013)
*Fraxinus americana*	Leaves	Growing	(12.5)	2.0	Ordonez and Olff (2013)
*Juniperus virginiana*	Leaves	Growing	(10.0)	1.6	Ordonez and Olff (2013)
*Ligustrum vulgare*	Leaves	Growing	(11.9)	1.9	Ordonez and Olff (2013)
*Lonicera japonica*	Leaves	Growing	(15.6)	2.5	Ordonez and Olff (2013)
*Rhamnus cathartica*	Leaves	Growing	(14.4)	2.3	Ordonez and Olff (2013)
*Rubus* sp.	Leaves	Growing	(13.8)	2.1	Ordonez and Olff (2013)
*Ulmus americana*	Leaves	Growing	(11.9)	1.9	Ordonez and Olff (2013)
*Acer saccharam*	Stem	Winter	(7.5)	1.2	Tripler et al. (2002)
*Fagus grandifolia*	Stem	Winter	(6.9)	1.1	Tripler et al. (2002)
*Fraxinus americana*	Stem	Winter	(5.6)	0.9	Tripler et al. (2002)

Few studies have investigated the contribution of invasive plants to the diets of deer. In their review, Parker *et al.* (2006) found that native herbivores generally reduce cover or the biomass of invasive plant species, consistent with the biotic resistance hypothesis (Elton 1958) that native species impede invasion, but this review included no studies from the temperate deciduous forest. In contrast, the enemy release hypothesis, which proposes that the success of invasives is due to reduced impact of natural enemies in the introduced range, predicts that herbivory by native generalists on invasive plants would be low (Colautti *et al.* 2004). While some invasive plants in eastern US forests are avoided by deer, others are preferred over some native species (Averill *et al.* 2016). One invasive shrub with ELP, *Ligustrum sinense* (Chinese privet), was an important part of the white-tailed deer diet in fall and winter during the years of acorn scarcity in Georgia, southeastern USA (Stromayer *et al.* 1998), and the vine *Lonicera japonica*, has long been planted as deer forage (Stransky 1984).

Smith’s (2013) hypothesis assumes that deer consumption of an invasive plant is associated with periods of ELP, that the invasive comprises a substantial component of deer diet, and that during periods of ELP the invasive is more nutritious than native plants. To test these assumptions, we studied the extent and temporal pattern of deer browse on *L. maackii* in an area with an extensive invasion of this shrub. We tested the seasonal component of this hypothesis by quantifying browse monthly over a 12-month period. We assessed the contribution of *L. maackii* to deer diets by estimating the mass of *L. maackii* browsed and how much of annual deer food consumption this comprised. Finally, we assessed whether *L. maackii* provided a nutritious food for deer by measuring the percent nitrogen in leaves and first-year stems and comparing these to published values for other available plants.

## Methods

### Study species

*Lonicera maackii* (Rupr.) Herder, Caprifoliaceae, Amur honeysuckle, is a large shrub introduced to North America in 1898 from northeastern Asia, and promoted for landscaping and erosion control (Luken and Thieret 1995). It now occurs in nearly all eastern and central states of the USA and is regulated as invasive in eight of those states (EDDMapS 2016). It expands leaves earlier in the spring (McEwan *et al.* 2009) and retains them later in the fall (Wilfong *et al.* 2009) than native deciduous woody plants.

Herbivory by invertebrates on *L. maackii* is very low (Lieurance and Cipollini 2011). However, Guiden *et al.* (2015) reported that deer browse occurred on 62 % of *L. maackii* branches during late fall/early winter. Two invasive congeners, the shrub *L. morrowii* and the vine *L. japonica*, are palatable to deer (Stransky 1984, Averill *et al.* 2016).

Negative effects of *L. maackii* on native plants have been inferred from lower abundance and species richness in stands or sites that are invaded compared to those not invaded (Hutchinson and Vankat 1997, Collier *et al.* 2002, Hartman and McCarthy 2008). Similar negative effects were manifest in field experiments (Gould and Gorchov 2000, Gorchov and Trisel 2003, Miller and Gorchov 2004, Hartman and McCarthy 2004, Orrock *et al.* 2015). There is also some evidence for allelopathy (Dorning and Cipollini 2006) and competition for soil water (Pfeiffer and Gorchov 2015).

### Study area

We studied white-tailed deer seasonal browse on *L. maackii* twigs and nutritional quality of *L. maackii* on 30 transects across the Miami University Natural Areas (MUNA) in southwestern Ohio (39° 29′ – 39° 31′ N, 84° 42′ – 84° 43′ W). MUNA totals 6.98 km^2^ and is comprised of patches of mature and young forests and successional fields abandoned from row crops and cattle pasture (Medley and Krisko 2007) located close to the University campus. *Lonicera maackii* is the dominant woody plant in the understory of the forests, with stem basal area ranging from 0.85 to 2.38 m^2^/ha in representative plots.

We distinguished three habitats utilized by deer: (1) *Juniperus*-dominated forest interior, (2) deciduous forest interior, and (3) forest/field edge. In this region, stands dominated by *J. virginiana* are successional, those within MUNA were abandoned from agriculture between 1950 and 1976 (Medley and Krisko 2007, L.M. Gramlich and K.E. Medley unpubl. data). We digitized polygons of fields, deciduous forest interiors, and *Juniperus*-dominated forest interiors within the bounds of MUNA using 2012 aerial photograph layers of a basemap from World Imagery in ArcGIS 10.1. Forest/field boundaries were delineated, and forest/field edges were defined as 5 m buffers that extend from these boundaries into forests. We used the **calculate geometry** command under attributes table of ArcGIS 10.1 to estimate the area of MUNA comprised of each of the three habitat types.

**Figure 1.**
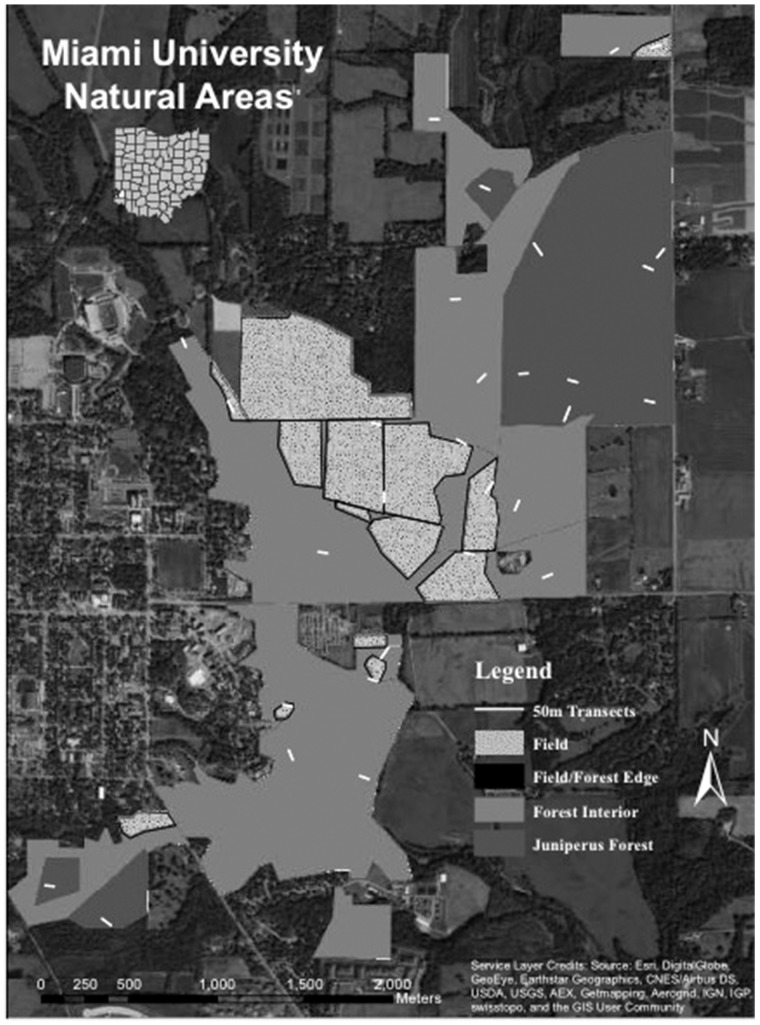
Map of Miami University Natural Areas showing field/forest edge, *Juniperus*-dominated forest interior, and deciduous forest interior habitats, and locations of the ten 50-m transects in each of these three habitats.

In each of the three habitats we randomly located 10–50-m transects (stratified random sampling) by specifying 10 starting points using the **generate random points** tool of ArcGIS 10.1, and replacing any points that fell on streams or steep slopes (Fig. 1). From each starting point, the direction of the transect was randomly selected from 4 cardinal and 4 primary intercardinal directions, excluding those directions that would result in the transect exiting the habitat. A 50 x 50 cm (0.25 m^2^) quadrat was placed every 5 m along each transect.

### Species composition of woody browse

In each of the three habitats, we determined the frequency of each woody species by sampling the 100 quadrats in May 2015. In each quadrant, a species was only scored if it had a woody branch with leaves within the height interval of 0.3–2.1 m above the ground, the height range of deer browse (Frelich and Lorimer 1985).

### Extent and seasonal pattern of deer browse

Deer browse on *L. maackii* was surveyed monthly over the course of one year (May 2015–April 2016) using the quadrats described above. For each quadrat, if *L. maackii* was present in the 0.3 to 2.1 m range, we marked a branch with black Sharpie in May 2015 and scored each twig distal to that mark as browsed or unbrowsed. Deer browse can be distinguished from other types of browse because deer shred the bark without leaving teeth marks (Swift and Gross 2008). We distinguished twig browse from branch browse; twigs were defined as stems of first-year growth while branches were defined as older stems bearing twigs (Guiden *et al.* 2015). However, all observed browse was on twigs, except for a subset of the April 2016 browse events, so methods focus on twigs. The May 2015 census picked up all browse on new shoots. For each monthly census after that, we counted the number of newly browsed twigs on each of these marked branches; each browsed twig was marked with red Sharpie so it was not recounted. Using dial calipers we measured the remaining length and diameter of each *L. maackii* twig at the point it was browsed. We also counted the total number of *L. maackii* twigs within the 0.3–2.1 m height range in each quadrat. To calculate percent of *L. maackii* twigs browsed by deer each month in each habitat, we divided the browsed twigs in each month by the total initial number of twigs on marked branches.

In April 2016, new leafy twigs expanded on many of the ‘old’ (2015 growth) twigs on the marked *L. maackii* branches. Therefore, in that month’s census, we separately counted new browse on ‘old’ twigs and new (2016 growth) twigs.

To determine how browse intensity varies with height, each browsed twig was classified into one of four height classes (0.3–0.7; 0.7–1.2, 1.2–1.7, and 1.7–2.1 m).

### Contribution of *L.maackii* to deer diet

To address the contribution of *L. maackii* to deer diet, we estimated the monthly consumption of *L. maackii* by deer in MUNA, summed this for 12 consecutive months, and divided this by various estimates of the total mass of food consumed by deer in MUNA.

To estimate monthly consumption of *L. maackii* by deer in each habitat, we estimated the number of browsed *L. maackii* twigs per unit area, multiplied this by the average leaf mass and average stem mass of a browsed twig, multiplied each of these products by the area of habitat in MUNA, then summed these totals (Eqn. 1

(1) $$\text{Monthly}\;\text{Consumption}\;=\left(\frac{\sum_{i=1}^{N_{1}}\frac{b_{i}}{o_{i}}t_{i}}{N_{1}a}\right)\frac{m_{1}H_{1}}{1000}+\left(\frac{\sum_{i=1}^{N_{2}}\frac{b_{i}}{o_{i}}t_{i}}{N_{2}a}\right)\frac{m_{2}H_{2}}{1000}+\left(\frac{\sum_{i=1}^{N_{3}}\frac{b_{i}}{o_{i}}t_{i}}{N_{3}a}\right)\frac{m_{3}H_{3}}{1000}$$

b_i_= number of twigs browsed subsequent to the last census on a marked branch in quadrat i; o_i_= number of twigs observed on marked branch in quadrat i; t_i_= total number of twigs in quadrat i; a= quadrat area (0.25 m^2^); N_1_= number of quadrats in *Juniperus* forest =100; N_2_= number of quadrats in forest/field edge =100; N_3_= number of quadrats in deciduous forest interior =100; m_1_= average leaf or stem mass of a browsed twig in *Juniperus* forest (g); m_2_= average leaf or stem mass of a browsed twig in forest/field edge (g); m_3_= average leaf or stem mass of a browsed twig in deciduous forest interior (g); H_1 _ = _ _area of *Juniperus* forest in MUNA (m^2^); H_2 _ = _ _area of forest/field edge in MUNA (m^2^); H_3 _ = _ _area of deciduous forest interior in MUNA (m^2^).).

Twigs browsed per unit area was estimated by summing across quadrats the product of the total number of twigs in the 0.3–2.1 m height range x the proportion of twigs on the marked branch that were browsed since the last monthly census; this sum was divided by the number of quadrats (100) x quadrat area (0.25 m^2^). This quotient was then multiplied by the average leaf or stem mass of a browsed *L. maackii* twig for that habitat (see below paragraph) to estimate the leaf mass and stem mass of browsed *L. maackii* per unit area for that month. We then multiplied each of those products by the area of MUNA comprised of that habitat. Those three values were summed to estimate the total browse of *L. maackii* in MUNA that month.

In order to estimate the leaf and stem masses of browsed portion of twigs in each of the three habitats, we used a method similar to that of Schmitz (1990), who weighed comparable portions from unbrowsed twigs. We predicted the leaf and stem mass of each browsed twig using allometric regressions relating leaf and stem masses of clipped twigs to dimensions that could be measured on browsed twigs. The dimensions measured were the length and diameter remaining at the point the twig was browsed or clipped.

Since long shoots have different morphology than regular twigs, and there is a suggestion they experience higher deer browse (D. Lieurance, pers. comm.), we counted long shoots separately in terms of scoring browse events and in parameterizing allometric regressions. In July 2015, we collected 30 unbrowsed twigs and 20 unbrowsed long shoots in each habitat, sampling from shrubs near each transect. For each of these twigs and long shoots we measured both the remaining length (L) of each twig and the diameter (d) of each twig at the point it was clipped. For each sample, we separated the leaves from woody tissue and dried each at 65 °C for 3 days before weighing. Each of the four sets of dry masses (leaf or stem, twig or long shoot) was regressed on L and d. As twigs approximate cylinders, use of d^2^ gave a better fit than d for each of these multiple regressions. We confirmed that these regression equations (Table 2) accurately predicted the mass *of L. maackii* twigs and leaves, by regressing observed mass on predicted mass. For each of the four data sets the regression model was a good fit (*R*^2 ^>^ ^0.6, Martinod 2016).

**Table 2.** Regression equations and statistics to relate leaf and stem mass of clipped *L. maackii* twigs to dimensions that could be measured on twigs browsed by deer. L is the length remaining (cm) and d is the diameter (mm).

Regression equation	*P*-value	R^2^
Leaf mass = 0.0472282–0.0007413L + 0.1472752d^2^	< 2.163e-07	0.2973
Twig mass = −0.0061760–0.0003922L + 0.0345029d^2^	< 2.2e-16	0.6461
Long shoot stem = −0.0252378–0.0010255L + 0.06393d^2^	< 2.2e-16	0.8232
Long shoot leaf = 0.0297021–0.0005147L + 0.1250721d^2^	< 2.2e-16	0.7848

The leaf and stem mass of each browsed *L. maackii* twig or long shoot was estimated from its L and d as measured the month it was first scored as browsed and the appropriate equation (Table 2). For twigs browsed between December 2015 and March 2016 we only estimated stem mass because *L. maackii* was leafless.

In April 2016, there were new twigs that expanded, requiring us to distinguish three types of browse: (1) browse on new twigs, (2) browse on old twigs (2015 growth) that showed no new growth, and (3) browse on old twigs that left some new growth. To quantify browse on new twigs (type 1), we counted the new browsed twigs on each marked branch and for each of these measured L and d and then collected an unbrowsed new twig with the same L and d and obtained its dry mass of leaf and stem tissue. The mean dry masses of leaf and stem tissue for each habitat [**seeSupporting Information—Table S2**] was used these to estimate leaf and stem mass consumed on browsed new twigs.

For browse in April 2016 on old twigs we first scored whether or not any new (2016) stems and leaves remained on the browsed twig. If there was no new growth (type 2) we assumed browse occurred before new twig expansion and therefore estimated only ‘old stem’ mass from measures of L and d and the allometric equation (Table 2).

If there was new growth on a browsed old twig (type 3), we recorded its L and d, then collected an unbrowsed old twig with new growth with the same L and d, separated, dried, and weighed the old stem tissue, new stem tissue, and new leaves. For each habitat we calculated the mean mass of old stem, new stem, and new leaves for these samples [**seeSupporting Information—Table S2**] and used these in estimating the mass of each tissue consumed.

In order to estimate the total mass of food consumed by deer in MUNA, we used several different estimates of daily consumption by deer from the literature (Table 3) and multiplied by the estimated number of white-tailed deer in MUNA (Eqn. 2).

**Table 3.** Estimated daily dry mass intake of white-tailed deer from the literature (c in equation 2) and the corresponding estimate of annual deer diet comprised of *L. maackii* based on equations 1 and 2. Literature values for intake are based on captive deer in winter to early spring, except for the first line, which is based on wild deer in winter. The range in the diet composition corresponds to mean ±1 SE of estimates of deer density in MUNA from Barrett (unpubl.).

Daily Intake (kg)	Sex	Description	Source	Annual estimate of deer diet comprised of *L. maackii* (%)
3.37	Female	Estimated using average weight of adult wild does and daily digestible energy requirement for winter maintenance with white cedar browse	Ullrey et al. (1970)	16 % (14–19 %)
2.60	Female	Observation that deer did not consumed more than 2.60 kg of white cedar browse during feeding experiments	Ullrey et al. (1970)	21 % (18–25 %)
1.40	Female	Average daily consumption based on captive deer eating white cedar browse	Ullrey et al. (1970)	39 % (33–47 %)
1.666	Female	Estimated using Schmitz (1990) equation and known digestible energy of experimental foods	Berteaux et al. (1998)	33 % (28–39 %)
2.163	Male	Estimated using Schmitz (1990) equation and known digestible energy of experimental foods	Berteaux et al. (1998)	25 % (22–30 %)

We estimated deer density in MUNA as 14.0 ± 2.3 (SE)/km^2^, based on ten estimates (transects in five areas x two seasons (summer, winter)) for MUNA in 2013 made by Barrett (2014) using pellet-based distance sampling (Urbanek *et al.* 2012).

### Crude protein

To quantify the nutritional quality of twigs, we determined percent nitrogen (%N) in samples of *L. maackii* twigs without leaves (February 2016) and with leaves (May 2016) and multiplied by 6.25 (Berteaux *et al.* 1998) to estimate crude protein (CP). We obtained %N values from 120 samples of twig stems (20 samples x 2 seasons x 3 habitats) and 60 samples of leaves (20 samples x 3 habitats). In each of those two months, we clipped two twigs near each of the 10 transects in each habitat within the height, d, and L typical of *L. maackii* twigs browsed by deer (0.3–1.2 m above the ground, d of 0.5–1.0 mm, and L of 1–5 cm; Martinod 2016). Once we clipped each sample, we wrapped its clipped end in moist paper towel, placed it in a sealed plastic bag, breathed into the bag to enhance CO_2_ concentration, and placed the sealed bag into a dark icebox (Pérez-Harguindeguy *et al.* 2013).

In the lab, the leaves were separated from stems (May only) and both were dried at 60º C. Each dry sample was ground separately and placed into a desiccator. Subsamples of 3–5 mg for stems and 2–3 mg for leaves placed in tin capsules, sealed, and kept frozen until analysis. Percent N was determined with the carbon-nitrogen analyser, Thermo Scientific FLASH 2000 NC Analyzer, at the Center for Aquatic and Watershed Sciences (CAWS) at Miami University.

To estimate the %N in leafy twigs we calculated a weighted average using mean %N of spring stems and leaves and mean stem and leaf masses from the unbrowsed twig samples collected in July 2015.

### Statistical analyses

For each of the three habitats we tested whether there was a seasonal pattern of deer browse on *L. maackii* twigs with a Chi-Square Goodness of Fit test. For each habitat, the expected number of browsed twigs in month m (m  =  1 for May 2015 and m  =  12 for April 2016 ‘old’ twigs) was

(3) $$B_{e}\left(m\right)\;=\;\text{Ib}{\left(1-b\right)}^{\left(m-1\right)}$$

In Eqn 3, I is the total sample of twigs observed (the initial number of twigs on the marked branches) and b is the monthly browse rate assuming constant browse over the 12 months (exponential decay of unbrowsed twigs), which was calculated using

(4) $${\left(1-b\right)}^{12\;}=\;1-B_{T}$$

where B_T_ is the observed total % browsed after 12 months.

## Results

### Species composition of woody browse

**Figure 2.**
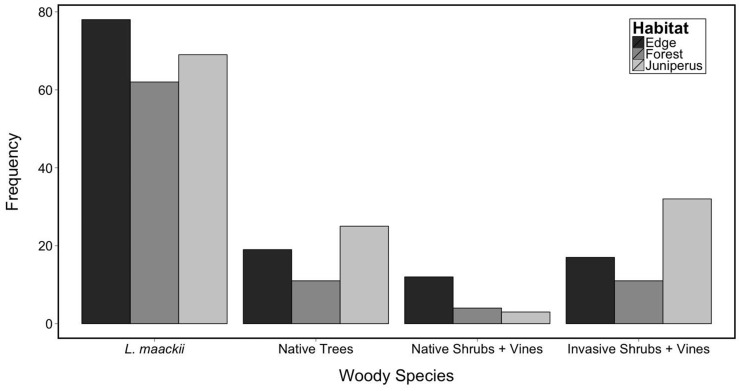
Frequency of woody species at deer browse height range (0.3–2.1 m) in each of three habitats in the Miami University Natural Areas. Species other than *L. maackii* were grouped into three categories: native trees, native shrubs and vines, and invasive (non-native) shrubs and vines. For frequencies of each species in each habitat see Supplemental Information Table A1.

A total of 25 woody species had leafy twigs within the 0.3–2.1 m height range [**see Supporting Information**]. The most frequently found woody species in all three of the habitats was *L. maackii* [Fig. 2, **seeSupporting Information—Table S1**]. Other woody species found at high frequency were *Vitis* spp. in field/forest edges, *Rosa multiflora* in deciduous forest interior, and *Fraxinus* spp., other *Lonicera* spp., and *Ligustrum* spp. in *Juniperus*-dominated forest habitats. In each habitat the frequency of *L. maackii* exceeded the sum of the frequencies of all other woody species combined (Fig. 2).

### Extent and seasonal pattern of deer browse

Deer browsed 719 out of 3258 twigs on the marked branches over 12 months (May 2015–April 2016). In addition, in April 2016 deer browsed 88 out of 3266 new twigs on these marked branches. The marked branches included only 9 long shoots (included in the sample of 3258), and deer browsed 4 of these.

**Figure 3.**
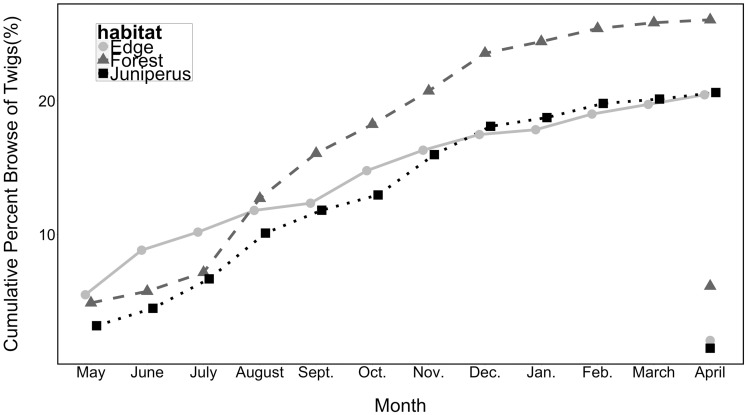
Cumulative deer browse on *L. maackii* twigs on marked branches by habitat type from May 2015 to April 2016. Sample size (number of twigs on marked branches) were 1110 for field-forest edge, 921 for deciduous forest, and 1227 for *J. virginiana*—dominated forest. In April 2016, we report browse on new shoots expanding on the marked branch as well as on the older cohort of twigs.

Deer browsed 0.2–6 % of *L. maackii* twigs per month [**seeSupporting Information—Fig. S1**]. In each of the three habitats, the observed number of twigs browsed per month differed significantly (*P* < 0.001) from that expected under the assumption of a constant monthly browse rate (Chi-Square Goodness of Fit test, X^2 ^ = ^ ^119.1, 129.1, and 78.4 for forest interior, edge, and *Juniperu*s-dominated forest habitats, df  =  11). Deer browse was moderate to high in spring and summer, moderate in fall, and low in winter [Fig. 3, **seeSupporting Information—Fig. S1**]. Monthly browse was greatest (> 4.5 % of twigs) in May 2015 for field/forest edge and in May, August, and April for forest interior habitats [**seeSupporting Information—Fig. S1**]. In *Juniperus*-dominated forests monthly deer browse never exceeded 3.5 % of *L. maackii* twigs [**seeSupporting Information—Fig. S1]**.

The cumulative percent of 2015 twigs browsed was highest in forest interior (26 %) and somewhat lower (20–21 %) in field/forest edge and *Juniperus*-dominated forest habitats (Fig. 3). When browse on new (April 2016) twigs borne on the marked branches is included, over the course of one year the cumulative browse was 32 % for forest interior vs. 22 % for the other two habitats.

**Figure 4.**
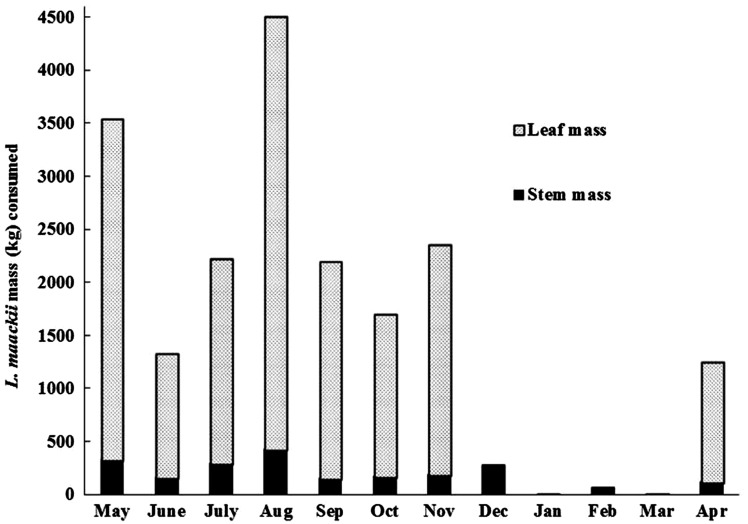
Estimated monthly leaf and stem masses of *L. maackii* consumed by white-tailed deer in the Miami University Natural Areas (MUNA), from Equation 1. Consumption per hectare is based on the 6.98 ha area of MUNA.

We estimated that deer in MUNA consumed a total of 17307 kg of *L. maackii* leaves (2479 kg/ha) and 2151 kg of *L. maackii* twig stems (308 kg/ha) over the course of 12 months. Consumption of leaves was > 1000 kg (> 150 kg/ha) each month except when the leaves were not available (December–March) and > 3000 kg (> 450 kg/ha) in May and August (Fig. 4). Deer consumed > 300 kg of stems in May and August (Fig. 4). Total consumption (leaves +  twigs) was high in the spring and summer and lowest in winter (Fig. 4). Consumption peaked in August (4505 kg), but deer also consumed > 3500 kg in May (Fig. 4).

### Estimation of contribution of *L.maackii* to deer diet

*L. maackii* was estimated to comprise 14–47 % of annual deer diet based on different deer daily food intake estimates from the literature (Table 3). The highest estimate is derived from the average consumption of captive does in winter and early spring and the energy content of white cedar browse (Table 3, Ullrey *et al.* 1970), whereas the lowest estimate comes from the estimated energy required by does to maintain body weight in winter, and the energy content of the same browse (Table 3, Ullrey *et al.* 1970).

### Nutritional quality

The mean percent nitrogen of *L. maackii* tissue was 1.60 % for winter stems, 0.87 % for spring stems, and 2.24 % for spring leaves, resulting in crude protein (CP) estimates of 10.0, 5.4, and 14.0 for these tissues (Table 1).

Based on the average stem mass and leaf mass of twigs collected in 2015 for parameterizing the allometric equations and %N in spring stems and leaves, we estimated leafy twigs averaged 2.07 % N, corresponding to 12.9 CP.

Literature values of CP ranged from 7.3 to 22.1 for leaves and 13.8 to 17.4 for leafy stems (Table 1). CP of other woody species available to deer in MUNA ranged from 5.6 to 7.5 for stems and 10.0 to 16.9 for leaves (Table 1).

## Discussion

Our findings provide support for key assumptions of the hypothesis that an invasive plant with extended leaf phenology impacts native plants via apparent competition (Smith 2013). We found that deer browsed on *L. maackii* throughout the year, but particularly in spring and summer, and estimated this invasive shrub comprised a large fraction of the annual food consumed by deer in the study area. Furthermore, twigs of *L. maackii* were more nutritious than twigs of other woody species available in the habitat during early spring, as they had higher protein content due to early leaf expansion during a time of protein limitation for deer.

### Seasonal pattern of deer browse

Though we documented deer browsed on *L. maackii* throughout the year, we estimate the mass consumed was high in early spring (April, May) and summer (August). (Note that censuses for browse took place in the middle of each month and documented browse that occurred during the month-long period since the previous census. The exception was May 2015 which picked up all browse on new shoots.) In spring 2015, *L. maackii* was the first species to begin leaf expansion, in mid to late April, followed by *Ligustrum* spp., which are also invasive. Bud break for native woody plants, such as *Cercis canadensis*, *Fagus grandifolia*, and *Acer saccharum*, occurred in early to mid-May (pers. obs.). In spring 2016, temperatures were warmer earlier and *L. maackii* began to break bud even sooner than usual (late March to early April) (pers. obs.). Because of its early leaf expansion, *L. maackii*’s presence in a forest has an impact analogous to an ‘early spring’ (Moen 1978), providing deer with a leafy, high protein food source when they would otherwise have access to only leafless, low protein browse. The peak *L. maackii* consumption in August may be explained by the mid-summer peak in energy consumption of lactating does, due to the energy demands of milk production (Moen 1978), or due to scarcity of herbaceous forage following senescence of many forest herbs.

While there is a period in late fall (typically November in our study site, Wilfong et al. 2009), when native woody plants have dropped their leaves and *L. maackii* still bears leaves, there was little deer browse on *L. maackii* during fall and winter. This contrasts with the seasonal patterns of deer consumption of *Ligustrum sinense* (Chinese privet), another ELP invader, in Georgia, southeastern USA (Stromayer *et al.* 1998). During years of acorn scarcity, *L. sinense* was an important part of the white-tailed deer diet in fall and winter (Stromayer *et al.* 1998). Our study did not fall within an acorn mast, so we attribute the low consumption of *L. maackii* to deer foraging on corn and soybean, which were cultivated near MUNA and thus available to the deer in our study. In landscapes comprised of forest and cropland, agricultural crops comprised a large fraction of deer diets (year-round in Illinois, Nixon *et al.* 1991; in summer, fall, and winter in Tennessee, Weckerly and Kennedy 1992). While deer consumption of soy is typically highest in the summer before this crop sets fruit (Colligan *et al.* 2011), deer eat corn from emergence through harvest, with peak use of corn fields from the tasseling-silking stage through harvest (Vercauteren and Hygnstrom 1998), which corresponded to July–October for Ohio in 2015 (NASS 2015). Deer will choose to eat agricultural crops over wild plants if the crops have higher CP (Dostaler *et al.* 2011). While agricultural lands did border Stromayer *et al.* (1998)'s study site, its larger size (2,138-ha compared to MUNA’s 698 ha) means that the crops were outside the home ranges of some of the deer. Additionally, winter deer browse on *L. maackii* may be lower than that on *L. sinense* (Stromayer *et al.* 1998) because the latter shrub is semi-evergreen (*L. maackii* is leafless in winter). The greater spring browse on *L. maackii* compared to *L. sinense* may be attributable to the availability of nutritious evergreen species in Stromayer *et al.*’s study site; the only evergreen browse available to deer in MUNA was *J. virginiana*.

### Contribution of *L.maackii* to deer diet

Our range of estimates for the proportion of annual food consumption comprised of *L. maackii* (14–47 %) reflects both the range of estimates for daily dry mass food consumption and estimates of deer density in our study area. Those density estimates were based on pellet-based distance sampling, a method shown to generate estimates similar to aerial surveys if accurate pellet decay and deposition rates are used (Urbanek *et al.* 2012). The density estimates used in this study were derived from Barrett (2014), who parameterized pellet decay rate from pellet groups observed in the same sites and seasons, but used average deposition rates from the literature. If actual deer densities were higher or lower than the range of densities we used, then the actual contribution of *L. maackii* to deer diet would be lower or higher (respectively) than our range of estimates. We plan to pursue an alternative method of quantifying the importance of *L. maackii* in deer diets in early spring, metagenomics analysis of deer fecal samples (Erickson *et al.* 2017).

### Nutritional quality

This study showed the leafy twigs of *L. maackii* in early spring were higher in protein than leafless woody stems, thus providing deer with a nutritious food source at this key time. Deer need protein in spring and summer for recovering from winter starvation, reproduction, lactation, growth, and maintenance (Berteaux *et al.* 1998, Pekins *et al.* 1998, Dostaler *et al.* 2011). While both leaves and stems of *L. maackii* had protein content that was moderate compared to those of leaves and stems of other woody species available to deer in MUNA (Table 1, Ordonez and Olff 2013), in early spring the protein content of leafy *L. maackii* twigs is much greater than that of stems of co-occuring native trees in leafless condition (Table 1, Tripler *et al.* 2002). However, once the native woody plants have leafed out, *L. maackii* leafy twigs are not a particularly nutritious food for deer; while we did not find data for the species common in our transects, most woody plants eaten by deer in south Texas had ‘browse’ (leaves and ends of twigs) with higher CP than *L. maackii* (Everitt and Gonzalez 1981).

### Potential for apparent competition

Although our findings support assumptions of Smith’s (2013) hypothesis of food-mediated apparent competition, they are not sufficient to demonstrate that *L. maackii* negatively impacts native plants through this mechanism. High deer populations negatively affect many native plant species (reviewed by Russell *et al.* 2001, Côté *et al.* 2004, McShea 2012, Habeck and Schultz 2015, Averill *et al.* 2017), including tree seedlings in MUNA (Peebles-Spencer *et al.* in prep.), to demonstrate apparent competition would require evidence that *L. maackii* elevated negative impacts of deer on native plants, due either to higher deer abundance or changes in deer feeding behavior. While Orrock *et al.* (2015) found evidence of this sort for small mammals (a negative impact on the native richness only where *L. maackii* fruits were present), it is difficult to use comparisons or large-scale experiments to test the effects of an invasive plant on deer impacts because deer move through the landscapes that are heterogeneous in vegetation and active management (McShea 2012) to access different foods in different seasons (Nixon *et al.* 1991), and they survive lean periods while losing body mass (Ullrey *et al.* 1970). It should be more feasible to compare deer impacts on native plants in sites with vs. without *L. maackii*, but a study design would need to overcome the challenges of vegetation and management heterogeneity over the landscapes travelled by individual deer.

While we do not have direct evidence that *L. maackii* invasion elevates deer populations, as Smith and Hall’s (2016) model predicts for ELP invasives, we suggest this is likely based on our finding that deer obtained a large fraction of their annual food budget from this shrub, including high protein twigs in early spring. One piece of evidence that is consistent with such a population response is the significantly higher density of deer fecal pellets in Missouri forest patches invaded by *L. maackii* (Allan *et al.* 2010). Although the density of fecal pellets may simply reflect spatial patterns of deer activity, rather than abundance, this could still result in apparent competition if deer browsed more on native plants in patches with invasive shrubs.

Most of the studies demonstrating invasive plants impacting native plants by ‘apparent competition’ really only document indirect amensalism (Chaneton and Bonsall 2000), as they document an indirect effect of the invasive on the native, but not vice-versa. However, in our system we have documented negative effects of deer on *L. maackii* as well. In MUNA plots where deer were excluded for five years, *L. maackii* shrubs had greater stem basal area growth than in paired deer-access plots (JR. Peebles-Spencer, C.M. Haffey, and D.L. Gorchov, unpubl. data). Furthermore, the greater basal area was manifest within the size class of shrubs where most branches were low enough to be accessible to deer (largest stem < 3 cm diameter), and greater cover was manifest within the heights of deer browse but not higher or lower (J.R. Peebles-Spencer, C.M. Haffey, and D.L. Gorchov, unpubl.). This negative effect of deer on *L. maackii* growth likely contributes to the experimental finding that the combined negative effects of deer and *L. maackii* on native plants are often less than additive (Christopher *et al.* 2014, Orrock *et al.* 2015, Loomis *et al.* 2015, Peebles-Spencer *et al.* in prep.). 

### Is deer consumption of invasive plants prevalent?

The importance of *L. maackii* to deer diets manifest in our results may be an artefact of the high frequency of this invasive shrub, and low frequency of other woody species, in the height range accessible to deer at our site. Our lab is currently investigating how the extent of browse on *L. maackii* depends on these abundances as well as on deer density. Based on the hypothesis that deer browse on *L. maackii* because it has leaf twigs in early spring, we expect this shrub will be a favored spring browse even where native woody plants are more common, but it will be a minor component of summer diets. Early in an invasion, while *L. maackii* shrubs are still sparse, we expect the proportion of its twigs browsed by deer would be very high, as they would be a low frequency but highly nutritious food in the early spring. If this browse is sufficient to prevent *L. maackii* from fruiting and from growing above the deer browse height, high density of this native herbivore during this early stage could actually impede invasion, consistent with the biotic resistance hypothesis (Elton 1958).

Generalization is not yet possible regarding herbivore impacts on invasive plants with ELP in North American deciduous forests. While in many Midwest US forests *L. maackii* is the most prevalent invasive shrub, many other invasive plants in these forests also expand leaves earlier in the spring than native plants (Smith 2013, Harrington *et al.* 1989, but see Fridley 2012). Some of these ELP invasive species rank high in feeding preference of deer (*Elaeagnus umbellata, Ligustrum vulgare, Lonicera morrowii*) while others do not (*Alliaria petiolata*, *Berberis thunbergii*; Averill *et al.* 2016). Even invasives of lower palatability may be important components of generalist diets if their ELP makes them available (i.e. bearing leaves) when native plants are leafless (Smith and Hall 2016).

## Conclusions

We estimated that *L. maackii* comprised 14–47 % of the annual food consumption by white-tailed deer in the Miami University Natural Areas, a site where this invasive shrub is more frequent in the deer browse height range than all other woody plants combined. This browse occurred year-round, but was great in early spring and late summer. Because of its early leaf expansion, *L. maackii* provides leafy twigs in the early spring, and these have higher (12.9 %) crude protein than leafless twigs of co-occurring native woody plants. These findings provide evidence for key assumptions of the hypothesis that shrubs with early leaf expansion, such as *L. maackii*, impact native plants via apparent competition.

## Supplementary Material

Supplementary Data

## Supporting Information

The following additional information is available in the online version of this article —

**Table S1.** Lists the percentage frequency of each woody species in each habitat type.

**Table S2.** Lists the average dry mass of stem and leaf tissue collected in April 2016 to correspond to browsed portions of old and new twigs in each of the habitats.

**Figure S1.** Shows monthly deer browse on *Lonicera maackii* twigs on marked branches in each habitat type.
