# Supplementary material for: White-tailed deer browse on an invasive shrub with extended leaf phenology meets assumptions of an apparent competition hypothesis
Source: AoB Plants. 2017 Feb 13;9(2):plx006. doi: 10.1093/aobpla/plx006 (PMC5397397; doi:10.1093/aobpla/plx006)
Supplement: Supplementary Data [file plx006_Supp.docx]

**Table S1.** The percentage frequency of each woody species in each habitat type. Each value is the percentage of the 100 0.25 m^2^ quadrats in which leafy branches of the species were present within the deer browse height range (0.3-2.1 m).

| **Species** | **Field/Forest Edge** | **Forest Interior** | ***Juniperus* Forest** |
| --- | --- | --- | --- |
| Invasive Shrubs & Vines  *Celastrus orbiculatus* | 5 | 0 | 2 |
| *Elaeagnus umbellata* | 2 | 0 | 5 |
| *Ligustrum* spp. | 5 | 3 | 11 |
| *Lonicera* spp. | 1 | 3 | 10 |
| *Lonicera japonica* | 0 | 0 | 1 |
| *Lonicera maackii* | 78 | 62 | 69 |
| *Rhamnus cathartica* | 1 | 0 | 0 |
| *Rosa multiflora* | 3 | 5 | 3 |
| Native Trees  *Acer negundo* | 6 | 2 | 0 |
| *Acer nigrum* | 1 | 0 | 0 |
| *Acer saccharum* | 0 | 2 | 1 |
| *Aesculus glabra* | 0 | 4 | 0 |
| *Celtis occidentalis* | 1 | 0 | 0 |
| *Cercis canadensis* | 0 | 0 | 1 |
| *Fagus grandifolia* | 0 | 1 | 0 |
| *Fraxinus* spp. | 4 | 2 | 19 |
| *Juniperus virginiana* | 2 | 0 | 3 |
| *Liquidambar styraciflua* | 0 | 0 | 1 |
| *Platanus occidentalis* | 1 | 0 | 0 |
| *Ptelea trifoliata* | 1 | 0 | 0 |
| *Ulmus americana* | 3 | 0 | 0 |
| Native shrubs & vines  *Rubus* spp. | 1 | 1 | 0 |
| *Viburnum dentatum* | 0 | 0 | 2 |
| *Viburnum prunifolium* | 0 | 3 | 1 |
| *Vitis* spp. | 11 | 0 | 0 |

**Table S2**. Average dry mass (g) of (A) old stem, new stem, and new leaf samples collected in April 2016 to correspond to browsed portions of old twigs (2015 growth) that had new growth on them, and (B) new growth twigs collected to correspond to browsed portions of new growth twigs in April 2016, in each of three habitats.

| **Browse type** | **Tissue Type** | **Field/Forest Edge** | **Forest Interior** | ***Juniperus* Forest** |
| --- | --- | --- | --- | --- |
| A Old twig | Old stem | 0.017 | 0.040 | 0.012 |
|  | New stem | 0.020 | 0.009 | 0.016 |
|  | New leaves | 0.161 | 0.113 | 0.112 |
| B New twig |  |  |  |  |
|  | New stem | 0.005 | 0.003 | 0.005 |
|  | New leaves | 0.059 | 0.040 | 0.051 |

**Figure S1.** Monthly deer browse on *L. maackii* twigs on marked branches by habitat type from May 2015 to April 2016. In April 2016, we calculated percent deer browse separately for 2015 stem growth (old) and 2016 stem growth (new).
